# Normative scores and clinical cut‐offs of the Cyclothymic–Hypersensitive Temperament Questionnaire in adolescence

**DOI:** 10.1002/jcv2.70126

**Published:** 2026-04-16

**Authors:** Anna Pezzella, Vincenzo Paolo Senese, Gabriele Masi, Annarita Milone, Giulia Mutti, Pietro Buono, Gianluca Sesso, Carmela Bravaccio, Raffaella Iuliano, Gennaro Catone, Simone Pisano

**Affiliations:** ^1^ Department of Psychology University of Campania “Luigi Vanvitelli” Caserta Italy; ^2^ Developmental Psychiatry and Psychopharmacology Unit IRCCS Stella Maris Foundation Pisa Italy; ^3^ Directorate General of Health Naples Italy; ^4^ IMT School for Advanced Studies Lucca Italy; ^5^ Department of Translational Medical Sciences University “Federico II” of Naples Naples Italy; ^6^ Department of Gynecology, Neonatology and Intensive Neonatal Care ASL Napoli 1 Naples Italy; ^7^ Department of Educational Psychological and Communication Sciences “Suor Orsola Benincasa” University Naples Italy

**Keywords:** adolescence, age‐ and sex‐adjusted normative references, bipolar spectrum disorders, clinical cut‐offs, cyclothymic–hypersensitive temperament

## Abstract

**Background:**

The Cyclothymic–Hypersensitive Temperament (CHT) is a multidimensional, transdiagnostic affective disposition characterized by mood instability, interpersonal sensitivity, heightened emotional reactivity and impulsive behaviors. The CHT Questionnaire (CHTQ) currently lacks of normative references and empirically derived thresholds.

**Methods:**

Study 1 established age‐ and sex‐adjusted normative scores and derived percentiles, tolerance limits, and Equivalent Score (ES)–based risk categories. Study 2 tested the clinical relevance of these norms in 196 adolescents with bipolar spectrum disorders and propensity score–matched controls, examining group differences, ES‐based risk distributions, and disorder‐specific thresholds.

**Results:**

In Study 1, regression analyses showed a significant age‐by‐sex interaction for Total and subscale scores. Age was positively associated with Total in females (*R* ^2^ = 0.029) and negatively in males (*R* ^2^ = 0.009). The same pattern emerged for Impulsiveness/Emotional Dysregulation (IED; females: *R* ^2^ = 0.029; males: ns) and Moodiness/Hypersensitivity (MHS; *R* ^2^ = 0.017 both sexes), supporting age‐ and sex‐specific norms. Normative values and ES‐based classifications enhanced score interpretability. Moderate‐ and high‐risk thresholds were 15.32 and 17.24 for Total, 6.67 and 7.70 for IED, 9.80 and 10.87 for MHS. In Study 2, bipolar adolescents showed higher adjusted Total and IED than controls (Total: *t* = −3.33, *p* < 0.001, *d* = 0.34; IED: *t* = −4.42, *p* < 0.001, *d* = 0.45), with no MHS differences. Logistic regression showed Total (odds ratio [OR] = 1.08, *p* = 0.001) and IED (OR = 1.20, *p* < 0.001), but not MHS, predicted bipolar status. Receiver Operating Characteristic (ROC) analyses showed modest discrimination for Total (Area Under the Curve [AUC] = 0.61; cut‐off = 13.50) and IED (AUC = 0.63; cut‐off = 4.78), and chance‐level performance for MHS.

**Conclusion:**

Integrating age‐ and sex‐adjusted norms with risk categories and clinical thresholds, the CHTQ may support developmentally informed early risk stratification and longitudinal monitoring.

## Key Points

**What's known?**

- Cyclothymic–Hypersensitive Temperament (CHT) is a multidimensional, transdiagnostic vulnerability disposition associated with internalizing and externalizing outcomes in adolescence; however, the lack of normative references and interpretable thresholds has limited the applied use of the CHT Questionnaire (CHTQ).

**What's new?**

- This study provides the first age‐ and sex‐adjusted normative scores for the CHTQ in adolescence, yielding non‐parametric tolerance limits and Equivalent Score–based risk categories.
- Clinical validation of normative scores in adolescents with bipolar spectrum disorders shows that elevated Total and Impulsiveness/Emotional Dysregulation—but not Moodiness/Hypersensitivity—scores are associated with bipolar status, and disorder‐specific cut‐offs were identified.

**What's relevant?**

- Age‐ and sex‐adjusted norms reduce the risk of over‐ or under‐identifying CHT during adolescence, supporting the use of the CHTQ as a dimensional risk stratification tool for early identification and longitudinal monitoring of affective vulnerability.

## BACKGROUND

The Cyclothymic‐Hypersensitive Temperament (CHT) has been described as a multidimensional affective disposition encompassing mood instability, interpersonal sensitivity, heightened emotional reactivity and impulsive or dysregulated behaviors (Hantouche et al., 2001; Kochman et al., 2005; Pisano et al., 2020). Its core features, emotional hyperreactivity and heightened sensitivity to social contexts, extend beyond transient mood fluctuations, reflecting a pervasive affective instability that influences both subjective emotional experience and interpersonal functioning (Akiskal et al., 1979; Perugi et al., 2017; Pisano et al., 2022).

According to several theoretical perspectives (Perugi et al., 2017), these temperamental affective characteristics are insufficiently captured by categorical nosography (as in the DSM‐5‐TR), which prioritizes episodic manifestations and fails to account for more stable or less specific affective configurations (Perugi & Akiskal, 2002). Such limitations support a dimensional and developmentally sound framework that conceptualizes affective temperaments as initial and subclinical manifestations of emotional vulnerability along a continuum bridging normative variability and psychopathological outcomes (Ghaemi & Dalley, 2014; Perugi & Akiskal, 2002; Perugi et al., 2017).

Within this broader theoretical framework, the empirical operationalization of affective temperaments—depressive, anxious, irritable, hyperthymic, and cyclothymic—was formalized through the Temperament Evaluation of Memphis, Pisa, Paris, and San Diego (TEMPS; Akiskal & Akiskal, 2005) and later refined into its self‐administered version, the TEMPS‐Autoquestionnaire (TEMPS‐A; Akiskal et al., 2005).

The CHT Questionnaire (CHTQ; Hantouche et al., 2001; Kochman et al., 2005) was developed as a child and adolescent adaptation of the cyclothymic dimension of the TEMPS‐A, expanding the original subscale with four additional items and reformulating its content in age‐appropriate language, resulting in a 25‐item self‐report measure for youth aged 7–18 years. Each item is presented as a brief descriptive statement and is rated using a dichotomous (“yes/no”) response format, indicating the presence or absence of the feature described. Since its development, the CHTQ has been adapted and applied in several cultural contexts, including Malaysian (Khodarahimi et al., 2011), French (Goutaudier et al., 2014), Chinese (Yuan et al., 2015), and Italian (Masi et al., 2018; Pisano et al., 2020). However, the psychometric properties of the scale have not been comprehensively examined across these adaptations. For example, to our knowledge, evidence on the factorial structure derives primarily from Italian samples (Pisano et al., 2020, 2022). These studies supported a correlated two‐factor structure and led to the refinement of a 22‐item Italian version following the removal of three poorly fitting items (items 2, 8, and 17 of the original scale). The first factor, named Impulsiveness/Emotional Dysregulation (IED), reflects affective instability, irritability, and impulsive or poorly regulated reactions; whereas the second factor, named Moodiness/Hypersensitivity (MHS), captures interpersonal sensitivity, rejection vulnerability, and emotional hyperreactivity (Pisano et al., 2020). In addition, the 22‐item version of the CHTQ showed solid psychometric properties and measurement invariance across sex, age, and across patients and controls (Pisano et al., 2020, 2022). Moreover, recently, Pezzella et al. (2025) extended this evidence by confirming the factorial structure and demonstrating its longitudinal measurement invariance, showing that the CHTQ consistently captures the same latent construct across developmental stages (pre‐adolescence to adolescence).

As regards the clinical relevance of the CHTQ, existing studies suggest that it can help to identify clinically meaningful risk phenotype, as its scores has been observed to be associated with a broad spectrum of internalizing as well as externalizing maladaptive outcomes—including depression, anxiety, suicidality, non‐suicidal self‐injury, impulsivity and behavioral problems (Kochman et al., 2005; Masi et al., 2018, 2023; Pezzella et al., 2025; Pisano et al., 2022). Recent longitudinal evidence showed that higher CHTQ scores are prospectively associated with increases in emotional and behavioral difficulties over time, reinforcing the construct's stability and its potential relevance for emerging psychopathology (Pezzella et al., 2025). Moreover, data showed that distinct patterns of association have been observed between each of the two CHTQ dimensions and different psychopathological profiles. In particular, the MHS factor was primarily associated with internalizing symptoms, whereas the IED factor showed stronger links with externalizing difficulties (Pisano et al., 2022).

Taken together, these findings are consistent with developmental perspectives suggesting that heightened affective reactivity and poor emotion regulation serve as foundational vulnerability markers for subsequent maladjustment (Masi et al., 2021). This emerging body of evidence supports the conceptualization of CHT as a transdiagnostic vulnerability disposition that may be particularly relevant for the early identification of psychopathological risk. However, fully realizing this potential in applied settings requires the availability of normative data and clinically interpretable scores, which are currently lacking for the CHTQ in all versions. In the absence of population‐based norms, derived through appropriate standardization procedures (Capitani & Laiacona, 2017), and empirically informed criteria for determining clinically meaningful thresholds (Nahm, 2022), individual scores cannot be meaningfully situated within a developmental or diagnostic framework. This gap limits the instrument's capacity to delineate levels of risk and to support its application in clinical or preventive contexts.

From a methodological standpoint, traditional approaches to normative data typically rely on parametric models that assume normally distributed measurements and use deviations from the mean to distinguish typical from atypical values (Crawford & Howell, 1998). This approach requires stratifying the normative sample into discrete subgroups (e.g., by age or sex) to compute specific reference parameters. However, this reduces the number of observations available for each estimate, thereby undermining the statistical stability and accuracy of the resulting norms (Capitani, 1997). In addition, psychological measures, particularly in the case of psychopathological dimensions, rarely conform to normality assumptions; thus, enforcing uniform statistical thresholds may oversimplify individual variability and yield reference standards that lack ecological validity (Capitani, 1997).

To address these limitations, a non‐parametric regression‐based approach have been introduced to model the relationship between demographic variables and test scores as a continuous function, producing more stable and accurate reference values (Aiello & Depaoli, 2022; Capitani & Laiacona, 2017). In this approach, raw scores are adjusted using regression equations that incorporate the estimated influence of relevant demographic factors, resulting in a single adjusted‐score distribution to which the same reference standards can be applied across all individuals. This adjusted distribution is then used to compute non‐parametric TLs, which define the widest interval expected to include a specified proportion of cases (Aiello & Depaoli, 2022); based on which the Equivalent Scores (ES) can be computed, which provide an ordinal classification method for interpreting Adjusted Scores (AS) (Capitani & Laiacona, 1997).

In clinical context, beyond population‐based norms, clinically meaningful cut‐off values are also required to support diagnostic decision‐making (Sheldrick et al., 2015). Indeed, unlike purely normative benchmarks, clinical cut‐offs are derived through comparisons with a clinical reference group, thereby linking score to clinically relevant outcomes and enhancing their applicability for diagnostic and preventive purposes. Such cut‐off values are typically identified using Receiver Operating Characteristic (ROC) curve analysis (Metz, 1978; Nahm, 2022), which determines the threshold that best balances sensitivity (true‐positive rate) and specificity (true‐negative rate) across all possible decision boundaries.

Starting from these considerations, to bridge the gap between the CHTQ's psychometric properties and its clinical applicability, two complementary studies aimed at establishing normative reference values (Study 1) and empirically derived clinical cut‐offs (Study 2) for the Italian adolescent population were carried out.

## STUDY 1

### Research question and hypothesis

The aim of this study was to establish nonparametric normative reference for the CHTQ in the Italian adolescent population. Indeed, as described in the general introduction, although the Italian version of the 22‐item CHTQ scale demonstrates strong psychometric properties (Pezzella et al., 2025; Pisano et al., 2020, 2022), its clinical usefulness is limited by the lack of population‐based norms. This study therefore sought to generate age‐ and sex‐adjusted normative scores for the CHTQ total and subscale scores and to compute TLs and ES by adopting the non‐parametric regression‐based approach (Aiello & Depaoli, 2022; Capitani & Laiacona, 2017).

### Methods

#### Sample

Data for the present study were selected from a database obtained by merging previously collected datasets from a series of large‐scale epidemiological studies conducted across Italy on samples of preadolescents and adolescents of the general population (Catone et al., 2019, 2020; Muratori et al., 2021; Pisano et al., 2020; Sesso et al., 2021). From these datasets, individuals aged from 10 to 17 years with complete CHTQ item‐level data were considered and included in the merged database (*N* = 3267). As reported in the original studies, all participants completed the protocols during regular school hours, in classroom settings, under the supervision of trained psychologists or research assistants. In all cases, participation was entirely voluntary, data were collected anonymously, and a written informed consent was obtained from both parents or legal guardians and participants prior to participation. Ethical approvals and procedural details for each data collection wave are available in the corresponding publications (Catone et al., 2019, 2020; Muratori et al., 2021; Pisano et al., 2020; Sesso et al., 2021).

#### Procedure

After merging all databases, a stratified random sampling procedure was implemented to address the initial overrepresentation of adolescents aged 10–14 years (see Table 1) and to ensure as much as possible a balanced number of participants for each sex‐by‐age combination. The resulting final normative sample comprised 1115 adolescents aged from 10 to 17 years (*M* = 12.63, SD = 2.04), of whom 595 (53.4%) were female.

**TABLE 1:** Distribution of participants in the final normative sample by sex and age (*N* = 1115).

Age (years)	Females (n)	Males (n)	Total
10	86	100 (119)	186 (205)
11	100 (505)	100 (473)	200 (978)
12	100 (447)	100 (495)	200 (942)
13	100 (404)	100 (409)	200 (813)
14	62	58	120
15	52	21	73
16	48	20	68
17	47	21	68
**Total**	595 (1651)	520 (1616)	1115 (3267)

*Note*: Values in parentheses indicate raw frequencies from the original merged database prior to age–sex stratified random sampling (original *N* = 3267).

#### Measures

##### Demographic information

For each participant, age (years) and sex (dummy coded: male = 1, females = 0) were considered, as these demographic variables were available across all datasets included in the study.

##### Cyclothymic Hypersensitive Temperament Questionnaire (CHTQ)

The validated Italian 22‐item version of the CHTQ was used in this study (Pisano et al., 2020, 2022). In datasets in which the 25‐item version had originally been administered, only the items corresponding to the validated 22‐item version were extracted for analysis (Cronbach's *α* = 0.82). The scale employs dichotomous response options (“yes/no”) and provides scores on two correlated dimensions: Impulsivity/Emotional Dysregulation (IED; Cronbach's *α* = 0.71) and Moodiness/Hypersensitivity (MHS; Cronbach's *α* = 0.70).

#### Data analysis

To derive age‐ and sex‐adjusted normative scores for the CHTQ total and subscale scores (IED and MHS), raw scores were corrected using a regression‐based procedure specifically designed to remove the influence of demographic factors (Aiello & Depaoli, 2022; Capitani, 1997; Capitani & Laiacona, 2017). In the first step, alternative transformations of age were tested in regression models predicting the total CHTQ score; the transformation of the age variable that provided the best model fit, that is the highest observed *R* ^2^, was retained in the models used to remove the influence of demographic factors. Then, to find the best model for adjusting raw scores, for each outcome (Total, IED, MHS), four regression models were compared: sex only (Model 1); transformed age only (Model 2); sex and transformed age (Model 3); and sex, transformed age, and their interaction (Model 4). Model selection was based on the corrected Akaike Information Criterion (AICc), which offers a more reliable selection criterion than the standard AIC (Burnham & Anderson, 2004). AS were computed by subtracting from each participant's raw score the demographic effect estimated by the best‐fitting regression model (Aiello & Depaoli, 2022). In practice, the regression coefficients of the selected model were applied as correction factors to each individual's deviation from the sample mean on the relevant predictors. This procedure was applied independently to each of the CHTQ scores (total, IED and MHS). Although it was not one of the specific objectives of the work, given the theoretical relevance of the observed demographic influences, both main and interaction significant effects were examined in detail, with interaction terms further explored through simple slope analyses.

Once the AS were obtained, to provide reference values for their interpretation, percentile ranks, TLs, and ESs were calculated from the empirical distribution of the AS. A non‐parametric approach was preferred for the TLs, as it has been shown to offer greater robustness than classical parametric procedures (Aiello & Depaoli, 2022; Capitani & Laiacona, 2017). In particular, for each dimension, outer and inner tolerance limits (oTL and iTL) were estimated respectively as the lowest adjusted score above which no more than 5% of the normative sample performs, with 95% confidence (oTL), and as the highest adjusted score below which, with the same confidence level, at least 95% of the sample scores are lower (iTL). To classify AS along a clinically interpretable continuum of risk, ESs were then derived (Capitani & Laiacona, 1997). Starting from the non‐parametric TLs, the distribution of AS was partitioned into five ordinal categories: “high risk”, “moderate risk”, “low risk”, “very low risk” and “no risk.” Scores above the oTL were assigned to the “high risk” category. Scores below the median were classified as “no risk”. Whereas the range of scores between the oTL and the median was divided into three equal‐width intervals corresponding to the intermediate categories. This procedure was applied independently to the CHTQ total score and to each subscale (IED and MHS).

All statistical analyses were performed using software R (version 4.3.2), following the analytic syntax and workflow described by Aiello and Depaoli (2022).

### Results

#### Selection of the best transformation

The proportion of variance explained in the model considering alternative transformations of the age variable is reported in Table 2. Among all tested transformations, the inverse transformation of age (1/Age) accounted for the largest amount of variance in the CHTQ total score, (*R* ^2^ = 0.0037, *p* = 0.043) and was therefore selected for subsequent normative corrections of raw scores.

**TABLE 2:** Proportion of explained variance (*R* ^2^) for each transformation of the age variable (CHTQ total score).

Transformation	Function	*R* ^2^
Linear	(Age)	0.0034*
Quadratic	(Age)^2^	0.0031
Cubic	(Age)^3^	0.0027
Logarithm base 100	log_100_ (Age)	0.0033
Natural log	ln (Age)	0.0036*
Square root	√(Age)	0.0035*
Inverse	1/(Age)	0.0037*
Logarithm base 10	log_10_ (Age)	0.0036*

*p < 0.05.

#### Sex‐ and age‐adjusted scores

Regression models on raw scores were then compared to identify the best‐fitting model for adjusting CHTQ scores based on demographic variables. Table 3 summarizes the fit indices for the four models tested separately for each CHTQ outcome.

**TABLE 3:** Comparison between regression models for the prediction of raw scores as a function of the CHTQ dimensions.

Model	Variables	AICc	ΔAICc	*R* ^*2*^	Δ*R* ^*2*^
CHTQ total					
Model 1	Sex	6501.04	17.52	0.006**	—
Model 2	*t*Age	6504.07	20.56	0.004*	—
Model 3	*t*Age + sex	6500.22	16.70	0.009**	0.005*^a^
Model 4	*t*Age + sex + *t*Age × sex	6483.52	—	0.025***	0.016***^b^
CHTQ IED					
Model 1	Sex	5116.61	12.34	0.007**	—
Model 2	*t*Age	5119.54	15.27	0.005*	—
Model 3	*t*Age + sex	5111.24	6.97	0.014***	0.009**^a^
Model 4	*t*Age + sex + *t*Age × sex	5104.27	—	0.022***	0.007**^b^
CHTQ MHS					
Model 1	Sex	5452.87	14.92	0.039***	—
Model 2	*t*Age	5495.94	57.99	0.001	—
Model 3	*t*Age + sex	5454.75	16.80	0.039***	0.038***^a^
Model 4	*t*Age + sex + *t*Age × sex	5437.95	—	0.055***	0.016***^b^

*Note*: Sex coded as 0 = female, 1 = male.

Abbreviations: ΔAIC*c*, difference in AIC*c* from the best‐fitting model (i.e. Model 4); Δ*R* ^*2*^, difference in *R* ^*2*^ from the hierarchically lower model; AIC*c*, corrected Akaike's Information Criterion; CHTQ IED, impulsivity/emotional dysregulation subscale score; CHTQ MHS, moodiness/hypersensitivity subscale score; CHTQ Total, total score of Cyclothymic–Hypersensitive Temperament Questionnaire; tAge, inverse transformation of the age variable (1/Age).

^a^ Compared to Model 2.

^b^ Compared to Model 3.

*p < 0.05, **p < 0.01, ***p < 0.001.

Across all analyses, the model including the interaction between inverse age and sex (Model 4) yielded the lowest AICc and a significant increment of the *R* ^2^ if compared to the model with main effects only, indicating superior fit. Consequently, the parameters from Model 4—comprising inverse age, sex, and their interaction—were used to compute the adjusted scores (AS; see Table 4). Because age was modeled using its inverse transformation, it is worth to notice that a negative coefficient indicates a positive association with chronological age, whereas a positive coefficient reflects a negative association.

**TABLE 4:** Multiple regression analysis predicting CHTQ scores by sex, age (transformed), and their interaction (*N* = 1115).

Effect	CHTQ total	CHTQ IED	CHTQ MHS
	*b*	*b*	*b*
Intercept	15.20***	5.71***	9.48***
tAge	−58.17***	−30.62***	−27.55**
Sex	−8.30***	−2.38*	−5.91***
tAge × sex	93.74***	34.94**	58.80***

*Note*: Sex coded as 0 = female, 1 = male.

Abbreviations: CHTQ IED, impulsivity/emotional dysregulation subscale score; CHTQ MHS, moodiness/hypersensitivity subscale score; CHTQ Total, total score of Cyclothymic–Hypersensitive Temperament Questionnaire; *t*Age, inverse transformation of the age variable (1/Age).

*p < 0.05, **p < 0.01, ***p < 0.001.

**FIGURE 1:**
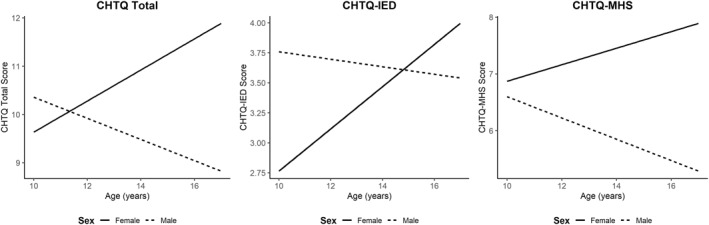
Simple slope plots illustrating the interaction between age and sex for the Cyclothymic–Hypersensitive Temperament Questionnaire Total score (left), the impulsiveness/emotional dysregulation subscale score (center), and the moodiness/hypersensitivity subscale score (right). Scores are modeled as a function of age, separately for females (solid lines) and males (dashed lines), showing differential developmental trajectories across sexes. For clearer interpretation of the interaction effect, the figure is based on the original age variable without transformation.

Regarding the interpretation of the parameters from the best‐fitting raw score correction models, the focus was primarily on the sex‐by‐age interaction, as it was significant across all three analyses and clarified the two main effects, which were also significant in each analysis. This indicates that the effect of each variable is moderated by the other. Specifically, age was considered the primary variable of interest, with sex treated as a moderating factor, although the same reasoning could be applied by reversing the roles of the two variables (see Figure 1). For the total CHTQ score, increasing age was associated with a small but significant increase in raw scores among females (*b*_*t*Age_ = −58.17, *p* < 0.001, *R* ^2^ = 0.029), whereas a weaker but still significant decrease was observed among males (*b*_*t*Age_ = 35.57, *p* = 0.035, *R* ^2^ = 0.009). For the IED subscale, a small but significant increase in raw scores with age emerged only among females (*b*_*t*Age_ = −30.63, *p* < 0.001, *R* ^2^ = 0.029), while the effect was non‐significant in males (*b*_*t*Age_ = 4.31, *p* = 0.640, *R* ^2^ < 0.001). For the MHS subscale, age was significantly and positively associated with raw scores in females (*b*_*t*Age_ = −27.55, *p* = 0.002, *R* ^2^ = 0.017), whereas a significant negative association was observed in males (*b*_*t*Age_ = 31.26, *p* = 0.003, *R* ^2^ = 0.017), indicating small effect sizes in both groups.

#### Adjusted scores

Descriptive statistics for the AS are reported in the Supporting Information (see Supporting Information S1: Table S1). The age‐ and sex‐specific correction factors that can be applied to the CHTQ total score and its subscales to get AS are presented in the Supporting Information (see Supporting Information S1: Table S2).

#### Population‐based reference values

To support the interpretability of AS for the CHTQ total and subscale scores (IED and MHS), percentile ranks were computed (see Table 5). These percentiles provide a descriptive framework for evaluating individual scores relative to the normative group. In addition to percentiles, inner and outer TLs (iTL and oTL) were also estimated. For the total CHTQ score, the iTL was 16.57 and the oTL was 17.23; for the IED subscale, 7.22 and 7.70; and for the MHS subscale, 10.58 and 10.87, respectively (see Table 5). Scores falling above the iTL can be considered statistically typical, whereas scores exceeding the oTL may indicate clinically relevant elevations. Values between the iTL and oTL represent a statistical “zone of uncertainty,” where interpretation should be made with caution and supported by converging information. Finally, to further enhance interpretability, ESs cut‐offs were also computed (see Table 5). In this way, observed AS can be classified on a five‐level categorical scale of risk (4 = “high risk”; 3 = “moderate risk”; 2 = “low risk”; 1 = “very low risk”; 0 = “no risk”), providing a uniform and clinically interpretable classification of individual measurements.

**TABLE 5:** Reference values for the interpretation of adjusted scores derived from the CHTQ.

References	CHTQ		
	Total	IED	MHS
Percentiles			
2.5th	1.2	−0.3	0.8
5th	2.5	−0.3	1.6
10th	3.8	0.5	2.8
20th	6	1	4
30th	7.5	1.7	5.2
40th	9	2.6	6
50th	10.09	3.03	6.76
60th	11.2	3.7	7.6
70th	12.5	4.7	8.2
80th	14.1	5.7	9
90th	15.6	6.7	10.2
95th	17	7.6	10.8
97.5th	17.8	7.7	11.2
TLs			
iTL	16.57	7.22	10.58
oTL	17.24	7.70	10.87
ESs			
0—no risk	<10.09	<3.03	<6.76
1—very low risk	10.09–12.63	3.03–4.75	6.76–8.40
2—low risk	12.64–15.31	4.76–6.66	8.41–9.79
3—moderate risk	15.32–17.23	6.67–7.69	9.80–10.86
4—high risk	≥17.24	≥7.70	≥10.87

Abbreviations: CHTQ Total, total score of Cyclothymic–Hypersensitive Temperament Questionnaire; ESs, equivalent scores; IED, impulsivity/emotional dysregulation subscale score; iTL, inner tolerance limit; MHS, moodiness/hypersensitivity subscale score; oTL, outer tolerance limit; TLs, tolerance limits.

## STUDY 2

### Research question and hypothesis

Building on the results of Study 1, this study examined whether the derived population‐based reference values also hold clinical relevance. To address this question, a sequential methodological approach, grounded in theoretical models and empirical findings linking CHT to bipolar symptoms in adolescence (Kochman et al., 2005; Masi et al., 2018; Parker et al., 2012; Perugi & Akiskal, 2002; Pisano et al., 2022) was adopted. First, in line with the cited literature, adolescents with bipolar spectrum disorders were considered as a possible benchmark for testing the clinical sensitivity of the CHTQ normative parameters. Consequently, data from individuals diagnosed with bipolar symptomatology were extracted from previously collected clinical datasets. Second, to derive a comparison group, a control sample of adolescents was extracted from previously collected datasets of community samples. In this latter case, adolescents showing clinically significant psychopathology based on standardized screening measures were excluded to ensure that the control group reflected a typical development. Then the two samples were matched on age and sex by using the Propensity Score Matching (PSM) procedure (Austin, 2011; Lee et al., 2010; Rosenbaum & Rubin, 1983). Once the final samples were obtained, the analyses were conducted to pursue two main objectives. The first was to examine the clinical validity of the normative values developed in Study 1 by testing whether they discriminated adolescents with bipolar disorder from matched controls. The second objective was to move beyond the population‐based norms and derive empirically based clinical cut‐offs for bipolarity through ROC curve analyses. We expected that adolescents with bipolar disorder would score higher on the CHTQ AS than matched controls, and that they would be more likely to exceed the normative threshold values established in Study 1. In addition, considering that CHT may represent a transdiagnostic vulnerability factor, by applying ROC analysis (Metz, 1978; Nahm, 2022), we expected to be able to identify clinical cut‐offs with sufficient specificity and sensitivity for the presence of bipolar disorder.

### Methods

#### Participants

##### Clinical sample

The clinical sample considered in this study was obtained from child and adolescent neuropsychiatry services across Italy by combining, into a single database, data that were already available and previously published with data already collected but not yet published. Specifically, in the first case, 143 adolescents with complete CHTQ item‐level data were extracted from three previously published clinical datasets (Masi et al., 2018, 2021; Sesso et al., 2021). To these, a further sample of 53 adolescents was added as part of an independent data collection conducted at the Child and Adolescent Neuropsychiatry Unit of the Department of Translational Medical Sciences, University of Naples “Federico II”, during routine clinical activity. This dataset was originally collected for different research purposes, and only CHTQ item‐level data were extracted and included in the present analyses. Regardless of the source, all patients were diagnosed using structured or semi‐structured clinical interviews, comprehensive psychiatric evaluations, and multidisciplinary assessments and a written informed consent was obtained from caregivers and adolescent. For additional methodological details and ethical approvals concerning the legacy datasets, readers can refer to the original publications (Masi et al., 2018, 2021; Sesso et al., 2021). Therefore, the final clinical sample consisted of 196 adolescents (49% female; age range 10–18; *M*_age_ = 14.01 years, SD = 1.90) diagnosed with bipolar disorder according to DSM‐5 criteria.

##### Control sample

Control data for the present study were selected from the same merged database created for the Study 1 but with different inclusion and exclusion criteria. In particular in this study, individual aged from 10 to 18 years with complete CHTQ item‐level data were considered and included as a starting control group (*N* = 3343). Moreover, to remove potentially undiagnosed or subclinical cases from the sample, an additional exclusion criterion was applied based on psychopathological screening. This latter step was carried out considering the presence of an observed score above the clinical cut‐off on one (or more) standardized and validated instruments present in the administered protocol: the Strengths and Difficulties Questionnaire (SDQ; Goodman, 1997), the Youth Self‐Report (YSR; Achenbach & Rescorla, 2001), and the Child Behavior Checklist (CBCL; Achenbach & Rescorla, 2001). Clinical thresholds were defined according to the normative indications available in the literature for each measure: a Total Difficulties score ≥20 for the SDQ (Goodman, 1997), and T‐scores >70 for the Total Problems scales of both the YSR and the CBCL (Achenbach & Rescorla, 2001). This resulted in the further exclusion of 262 cases, yielding a final reference database of 3081 adolescents in the control sample.

##### Matching procedure

To minimize confounding and strengthen the internal validity of group comparisons, a PSM procedure was applied to match the final clinical and control databases (Austin, 2011; Lee et al., 2010; Rosenbaum & Rubin, 1983). PSM is a widely recognized method in observational research for reducing selection bias by balancing observed covariates across groups and approximating key features of randomized controlled designs. In particular, a 1:1 nearest neighbor matching algorithm was implemented with exact matching on both age and sex (Austin, 2014). As a result, each clinical participant was matched with one control participant sharing the same demographic characteristics, resulting in a final matched database of 386 participants (age range: 10–18 years; *M*_age_ = 13.96 years, SD = 1.87) on which all analyses were conducted.

#### Measure

##### Demographic information

For each participant, age (years) and sex (dummy coded: male = 1, females = 0) were considered, as these demographic variables were available across all datasets included in the study.

##### Clinical classification

Diagnostic status (dummy coded: bipolar = 1, control = 0) was established according to the procedures adopted in the contributing clinical studies. For adolescents in the clinical group, bipolar disorder diagnoses were made in accordance with DSM‐5 criteria and supported by structured or semi‐structured clinical interviews, comprehensive psychiatric evaluation, and multidisciplinary assessment.

##### Cyclothymic‐Hypersensitive Temperament Questionnaire (CHTQ)

As in Study 1, the validated Italian 22‐item version of the CHTQ was used (Pisano et al., 2020, 2022). In datasets in which the 25‐item version had originally been administered, only the items corresponding to the validated 22‐item scale were extracted for analysis (Cronbach's *α* = 0.83). The scale employs dichotomous (“yes/no”) response options and provides scores on two correlated dimensions: Impulsivity/Emotional Dysregulation (IED); (Cronbach's *α* = 0.75) and Moodiness/Hypersensitivity (MHS; Cronbach's *α* = 0.70).

#### Data analysis

##### Adjusted scores

To ensure that the resulting scores were not confounded by age or sex, AS were computed for each participant for the CHTQ total and subscale scores. This was done using the regression‐based correction procedure defined in Study 1. Specifically, raw scores were adjusted by removing the modeled demographic effect, based on the regression coefficients previously estimated in the normative sample. To test the statistical significance of the differences between the mean AS (total, IED, and MHS) in the bipolar and control groups, independent‐samples *t*‐tests were conducted. Effect sizes were estimated using Cohen's *d*, providing a standardized index of the magnitude of between‐group differences. Finally, to further assess the discriminative validity of the CHTQ dimensions, three separate 2‐step hierarchical binary logistic regression models were estimated considering the clinical status (bipolar vs. control) as the dependent variable. In each model, in the first step sex and age were included as control variables, whereas in the second step one of the CHTQ AS computed (Total, IED, or MHS) was added. For each logistic regression model, performance was evaluated using two complementary indices derived from the observed–versus–predicted classification table: the overall classification accuracy, which expresses the proportion of correctly classified cases, and the predictive efficiency coefficient (*τ*_*p*_; Klecka, 1980), which quantifies the proportional reduction in classification error achieved by the model relative to chance‐level prediction.

##### Clinical validity of the ES

To evaluate the clinical validity of the ESs derived from the normative sample in Study 1, we tested whether these scores could discriminate between adolescents with and without bipolar disorder. For this purpose, the ES thresholds identified for the CHTQ scores, that is the total score and its two subscales, were applied to both samples. Each participant was classified into one of five risk categories: “no risk”, “very low risk”, “low risk”, “moderate risk”, or “high risk”. To test whether the distribution of risk profiles differed between the two groups, a Chi‐square (*χ* ^2^) test of independence was conducted for each CHTQ dimension. When significant, standardized residuals were extracted and examined to identify the specific risk levels contributing to overall group differences, highlighting whether any profiles were over‐ or underrepresented in each group.

##### Identification of clinical cut‐off

To identify clinically informative thresholds for classifying individuals according to bipolar status, for each CHTQ score, ROC curve analysis was performed using the AS as predictors and diagnostic status (bipolar vs. control) as the binary outcome. ROC analysis is a robust method for evaluating the discriminative capacity of a continuous measure by examining its sensitivity and specificity across varying thresholds (Metz, 1978; Nahm, 2022). In this context, sensitivity represents the proportion of true positives correctly identified, while specificity reflects the proportion of true negatives correctly classified. The ROC curve visually captures the trade‐off between these two parameters, and its Area Under the Curve (AUC) provides a global index of diagnostic accuracy, with values ranging from 0.5 (no discriminative ability) to 1.0 (perfect classification). For each CHTQ dimension, the optimal cut‐off was identified using Youden's *J* statistic (Youden, 1950), which maximizes the difference between the true positive rate and false positive rate, thereby identifying the threshold that yields the best balance between sensitivity and specificity.

### Results

#### Adjusted scores

The mean AS for the clinical and control groups, along with the results of the *t*‐tests, are reported in Table 6. As shown, adolescents with a bipolar diagnosis exhibited significantly higher ASs on both the CHTQ Total score, *t*(384) = −3.33, *p* < 0.001, and the IED subscale, *t*(384) = −4.42, *p* < 0.001, with corresponding effect sizes in the small‐to‐moderate range (*d* = −0.34 and −0.45, respectively). No significant difference emerged for the MHS subscale, *t*(384) = −1.30, *p* = 0.20.

**TABLE 6:** Mean adjusted CHTQ scores in clinical and control groups, with corresponding *t*‐test statistics, *p* values, Cohen's *d* effect sizes, and 95% confidence intervals.

	*M* (controls)	*M* (clinic)	*t (df)*	Cohen's *d*	95% CI (*d*)
CHTQ total	9.91	11.38	−3.33*** (384)	−0.34	[−0.54; −0.14]
CHTQ IED	3.51	4.60	−4.42*** (384)	−0.45	[−0.65; −0.25]
CHTQ MHS	6.40	6.75	−1.30 (384)	−0.13	[−0.33; 0.07]

Abbreviations: CHTQ IED, impulsivity/emotional dysregulation subscale score; CHTQ MHS, moodiness/hypersensitivity subscale score; CHTQ Total, total score of Cyclothymic–Hypersensitive Temperament Questionnaire; *M*, mean.

***p < 0.001.

As regards the discriminative capacity of the CHTQ dimensions, the three regression models (see Table 7) confirmed that over and above the control variables (sex and age), both the Total and IED scores significantly predicted group membership; whereas that the MHS subscale did not significantly contribute to discriminate the two groups (odds ratio [OR] = 1.05, *p* = 0.19). The model parameter analysis of the significant effect showed that each one‐point increase in the Total score was associated with an 8.2% increase in the odds of being classified in the clinical group (OR = 1.08, *p* = 0.001), and that each one‐point increase in the IED subscale was associated with a 20% increase in the odds (OR = 1.20, *p* < 0.001). The predictive efficiency coefficients (*τ*_*p*_) indicated modest discriminative contributions for the Total and IED scores (*τ*_*p*_ = 0.15 and *τ*_*p*_ = 0.19, respectively), whereas the value for the MHS score (*τ*_*p*_ = 0.04) reflected minimal improvement over chance (see Table 7). In terms of overall classification accuracy, the Total and IED models correctly classified 57.5% and 59.6% of cases, respectively, indicating a small but meaningful improvement over the 50% accuracy expected from random classification. By contrast, the MHS model yielded accuracy close to chance level (51.8%).

**TABLE 7:** Logistic regression coefficients predicting clinical status (bipolar vs. control) from adjusted CHTQ scores.

Predictor	*b*	SE	*z*	OR	*τ*_*p*_
Regression 1: CHTQ total					
Age	−0.014	0.056	−0.249	—	—
Sex	0.033	0.210	0.157	—	—
Total AS	0.079 ***	0.024	3.265	1.08	0.15
Regression 2: CHTQ IED					
Age	0.017	0.057	0.295	—	—
Sex	0.046	0.212	0.217	—	—
IED AS	0.185 ***	0.043	4.264	1.20	0.19
Regression 3: CHTQ MHS					
Age	−0.014	0.056	−0.246	—	—
Sex	0.010	0.207	0.046	—	—
MHS AS	0.052	0.039	1.320	1.05	0.04

Abbreviations: *b*, unstandardized coefficient; CHTQ IED, impulsivity/emotional dysregulation subscale score; CHTQ MHS, moodiness/hypersensitivity subscale score; CHTQ Total, total score of Cyclothymic–Hypersensitive Temperament Questionnaire; OR, odds ratio; SE, standard error; *τ*_*p*_, predictive efficiency coefficient.

***p < 0.001.

#### Clinical validity of the ES

As regards the clinical validity of the ES, the results of the Chi‐square (*χ* ^2^) tests confirmed the same pattern of results indicating that the distribution of ES‐based risk profiles differed significantly between bipolar and control participants for both the CHTQ Total score, *χ* ^2^ (4) = 22.69, *p* < 0.001, and the IED subscale, *χ* ^2^ (4) = 27.40, *p* < 0.001. No significant difference was observed for the MHS subscale, *χ* ^2^ (4) = 7.65, *p* = 0.10. As shown in Table 8, adolescents with a bipolar diagnosis were disproportionately represented in the “high”, “moderate”, and “low risk” categories, especially for the Total and IED scores. Conversely, control participants were more frequently classified in the “very low” and “no risk” categories. These patterns were further corroborated by the analysis of standardized residuals (Field, 2024), which exceeded the conventional |1.96| threshold in multiple categories for both the Total and IED scores, indicating statistically meaningful deviations from expected frequencies. In contrast, all residuals for the MHS subscale remained within the |0–1.96| range, supporting the absence of meaningful group differences on this dimension.

**TABLE 8:** Distribution of equivalent score risk categories across clinical and control groups for the CHTQ Total score and its subscales.

Risk level	Controls *N* (%) (std. res.)	Clinical *N* (%) (std. res.)	Total *N* (%)
CHTQ total			
High	4 (22%) (**−2.41**)	14 (78%) (**+2.41**)	18 (5%)
Moderate	15 (35%) (**−2.10**)	28 (65%) (**+2.10**)	43 (11%)
Low	24 (35%) (**−2.67**)	44 (65%) (**+2.67**)	68 (18%)
Very low	58 (60%) (**+2.22**)	39 (40%) (**−2.22**)	97 (25%)
No risk	92 (58%) (**+2.47**)	68 (42%) (**−2.47**)	160 (41%)
CHTQ IED			
High	7 (25%) (**−2.74**)	21 (75%) (**+2.74**)	28 (7%)
Moderate	22 (35%) (**−2.61**)	41 (65%) (**+2.61**)	63 (16%)
Low	19 (35%) (**−2.21**)	34 (65%) (**+2.21**)	53 (15%)
Very low	48 (55%) (+1.22)	38 (45%) (−1.22)	86 (22%)
No risk	97 (60%) (**+4.00**)	59 (40%) (**−4.00**)	156 (40%)
CHTQ MHS			
High	3 (25%) (−1.75)	9 (75%) (+1.75)	12 (3%)
Moderate	18 (54%) (+0.54)	15 (46%) (−0.54)	33 (9%)
Low	25 (41%) (−1.40)	35 (59%) (+1.40)	60 (16%)
Very low	47 (46%) (−0.81)	54 (54%) (+0.81)	101 (26%)
No risk	100 (55%) (+2.04)	80 (45%) (−2.04)	180 (46%)

*Note*: Values represent absolute frequencies, percentages within each risk level, and standardized residuals (in parentheses). Residuals exceeding ±1.96 reflect statistically meaningful deviations from expected frequencies.

Abbreviations: CHTQ IED, impulsivity/emotional dysregulation subscale score; CHTQ MHS, moodiness/hypersensitivity subscale score; CHTQ Total, total score of Cyclothymic–Hypersensitive Temperament Questionnaire.

#### Identification of clinical cut‐off

**FIGURE 2:**
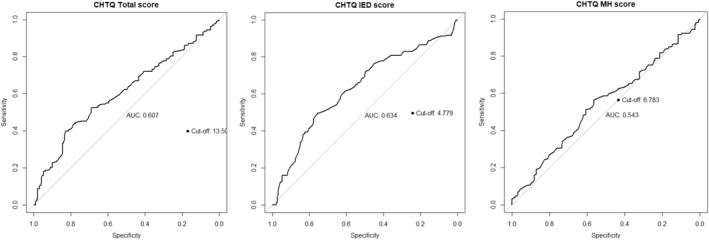
Receiver operating characteristic curves for the adjusted Cyclothymic–Hypersensitive Temperament Questionnaire Total score (left), impulsiveness/emotional dysregulation subscale score (center), and moodiness/hypersensitivity subscale score (right). Each panel displays the corresponding area under the curve and the optimal cut‐off point derived from the Youden index.

The ROC analysis for the CHTQ Total score yielded an AUC of 0.61 (95% CI: 0.55–0.66), indicating a modest discriminative ability of the score (Metz, 1978; Nahm, 2022). The optimal threshold identified via Youden's *J* (Youden, 1950) was 13.50, corresponding to a sensitivity of 40% and specificity of 83%. At this cut‐off, the positive predictive value (PPV) was 58%, and the negative predictive value (NPV) was 70%. These indices reflect the probability that an individual classified by the test as positive or negative truly belongs to the bipolar or non‐clinical group, respectively. A similar pattern was observed for the IED subscale, which demonstrated a slightly higher AUC of 0.63 (95% CI: 0.58–0.69). The optimal cut‐off of 4.78 yielded a sensitivity of 50% and specificity of 76%, with PPV of 60% and NPV of 67%. In contrast, the MHS subscale showed limited discriminative performance, with an AUC of 0.54 (95% CI: 0.49–0.60), not significantly different from chance. The optimal cut‐off of 6.78 produced balanced but low sensitivity and specificity (both 56%), with equivalent PPV and NPV (56%), indicating that the MHS score offers minimal clinical utility in distinguishing diagnostic status. The ROC curves for all three CHTQ dimensions are shown in Figure 2.

## DISCUSSION

The present work represents the first integrated attempt to provide both normative and clinical reference data for the CHT Questionnaire in Italian adolescents aged from 10 to 17 years. By combining large‐scale normative analyses with validation in a clinical sample of adolescents with bipolar spectrum disorders, this research moves the CHTQ beyond its original use as a descriptive temperament measure and toward a tool with concrete interpretative value in a clinical framework. In doing so, it bridges the gap between temperament research and applied adolescent psychiatry, offering empirical foundations for the developmental assessment of CHT.

Study 1 provided the first age‐ and sex‐adjusted normative benchmarks for the CHTQ. The most salient finding was the presence of a significant sex‐by‐age interaction. Specifically, females showed a pronounced age‐related increase in CHTQ scores, particularly for the Total and IED scores, with trajectories that became progressively steeper across adolescence; a similar, though less pronounced, upward trend was observed for the MHS score. In contrast, males displayed a different developmental profile, with scores tending either to remain relatively stable or to decline with increasing age, showing clear downward trends for the Total and MHS scores and minimal age‐related variation in IED. These divergent patterns extend previous evidence of sex differences in CHT (Masi et al., 2018, 2023; Pisano et al., 2020, 2022) by demonstrating that such differences evolve across adolescence rather than remaining static. More broadly, the observed patterns are consistent with developmental models documenting increasing socioemotional divergence between males and females during adolescence, linked to sex‐specific timing of neurobiological maturation and affective reactivity (Casey et al., 2010; Chaplin & Aldao, 2013; Steinberg, 2005). Converging neurobiological evidence supports a developmental interpretation of these patterns. Across adolescence, females exhibit earlier and stronger engagement of neural systems involved in emotion processing and regulation, and affective‐regulatory circuits appear to follow sex‐specific developmental configurations (Church et al., 2025; Pan et al., 2024). Consistently, emotional reactivity follows non‐linear trajectories more closely linked to pubertal stage than to chronological age, with females showing transient puberty‐related increases in affective sensitivity, whereas males tend to display more attenuated changes (Vijayakumar et al., 2019). Together, these converging lines of evidence suggest that the higher and progressively increasing CHTQ scores observed in adolescent females may reflect a more pronounced and developmentally timed expression of affective sensitivity and interpersonal reactivity, rather than heightened psychopathological vulnerability. Nevertheless, these interpretations must be tempered by the cross‐sectional nature of the present data, and longitudinal studies are required to determine whether the observed sex‐specific patterns reflect true individual developmental trajectories. From a measurement perspective, the robust sex‐by‐age interaction underscores the importance of age‐ and sex‐adjusted normative references, as failure to account for these factors may result in overestimation of vulnerability in adolescent females and underestimation in males.

Study 2 examined the clinical validity of the normative values established in Study 1 by testing their validity to differentiate adolescents with bipolar disorder from matched controls. Consistent with previous evidence linking CHT to bipolar‐spectrum features (Masi et al., 2018; Parker et al., 2012; Pisano et al., 2022), adolescents with a clinical diagnosis of bipolarity showed significantly higher CHTQ Total and IED scores, whereas no difference emerged for the MHS score. This suggests that the impulsiveness and emotional dysregulation captured by the IED score represent the component of CHT most closely aligned with bipolar status, while moodiness and hypersensitivity—although conceptually related to affective vulnerability—appear more broadly expressed in the general population and therefore less useful for distinguishing this specific group. The distribution of ES‐based risk categories further supported this interpretation. Clinical participants were overrepresented in the “high” and “moderate” risk categories for the Total and IED scores, whereas the MHS score showed no substantial group differences. Taken together, these findings align with prior work showing that CHTQ scores relate to a broad range of internalizing and externalizing difficulties (Pisano et al., 2022), while clarifying that only certain components—particularly those involving dysregulation and impulsive emotional reactions—show meaningful group discrimination. The ROC analyses offered a complementary perspective to the ES‐based classifications. Although both procedures relied on the same age‐ and sex‐adjusted scores, they served different analytic aims and therefore produced partly distinct patterns. ES categories, derived from the normative distribution established in Study 1, classify adolescents according to increasing levels of deviation from typical population values. Within this framework, the clinical group showed a clear concentration in the upper ES categories for the Total and IED scores, corresponding to the “moderate” and “high” risk categories (e.g., ≥17.24 for the Total score; ≥7.70 for IED). ROC analyses, by contrast, evaluate how well a continuous score distinguishes clinical from control cases across all possible thresholds. In line with this approach, the AUC values for the Total and IED scores were modest (AUC = 0.61–0.63), and the corresponding optimal cut‐offs (13.50 for the Total score and 4.78 for IED) reflected the best balance of sensitivity and specificity rather than deviation from normative patterns. For instance, although a Total score of 13.50 maximized Youden's J, it fell below the oTL of 17.24, indicating that the clinical optimal threshold does not necessarily coincide with the value identifying the upper 5% of the distribution at which scores are considered atypical. A similar pattern emerged for IED, where the ROC‐derived cut‐off of 4.78 fell within the “low‐risk” ES range (4.76–6.66), again reflecting the different logic underlying ROC‐based optimization. Together, these differences illustrate the distinct purposes of ES and ROC analyses. ES categories quantify how far an individual's score departs from what is typical for their age and sex, whereas ROC analyses quantify how well scores separate groups. Each method therefore provides complementary information: ES values index normative deviation, while ROC indices capture discriminative performance. Considered jointly, they offer a more complete understanding of how CHTQ scores function across normative and clinical contexts.

Importantly, the modest diagnostic accuracy observed in the ROC analyses is neither surprising nor inconsistent with the theoretical role of temperaments. Temperamental traits represent broad, transdiagnostic vulnerability factors rather than disorder‐specific markers (Akiskal & Akiskal, 2005; Pisano et al., 2020). From this perspective, the CHTQ should not be viewed as a diagnostic screening instrument for bipolar disorder. Instead, its value lies in identifying adolescents who deviate from normative temperamental patterns and who may warrant closer monitoring, particularly when elevated IED scores co‐occur with other clinical or contextual risk factors such as a family history of mood disorders, early emotional dysregulation, neurodevelopmental comorbidities, psychosocial adversity, or exposure to chronic stress, within a cumulative risk framework. When interpreted using age‐ and sex‐adjusted reference values, the CHTQ provides a clinically meaningful index of affective vulnerability that can support early detection and preventive efforts in adolescent mental health.

Taken together, these findings support a conceptualization of the CHT as a multidimensional profile, while also indicating that its components do not contribute equally to clinical differentiation. The impulsiveness and emotional dysregulation captured by the IED score showed the clearest association with bipolar status, consistent with evidence linking dysregulation‐prone temperamental traits to more severe affective presentations in youth. In contrast, the moodiness and hypersensitiveness reflected in the MHS score did not distinguish bipolar from control participants, suggesting that this component may represent broader interpersonal or emotional sensitivities that are not specific to bipolar disorder and may be more broadly distributed in the general adolescent population. Importantly, this pattern does not imply that MHS is clinically irrelevant; rather, it suggests that variability in this subcomponent may capture developmentally para‐physiological socioemotional fluctuations that are relatively common during adolescence and therefore reflect a more general affective vulnerability. During this developmental stage, heightened interpersonal sensitivity and mood variability represent normative phenomena linked to ongoing neurobiological maturation and increasing relational salience. In this context, MHS‐related traits may be widely expressed across both clinical and non‐clinical adolescents. For this reason, the dichotomous (presence/absence) response format of the CHTQ may be less sensitive to gradations in intensity, frequency, or functional impact, thereby limiting the subscale's ability to differentiate between developmentally typical affective sensitivity and clinically impairing manifestations. This differential profile across the two subcomponents of the CHTQ reinforces the importance of considering them separately, as consistently supported by previous psychometric investigations (Pisano et al., 2020, 2022). Treating Total, IED, and MHS scores as partially distinct indicators provides a more nuanced understanding of how different elements of CHT relate to clinical outcomes and may help clarify why only certain aspects show discriminative value.

A key consideration concerns how the interpretative risk thresholds derived in Study 1 relate to the sex‐specific clinical cut‐offs originally proposed by Pisano et al. (2020), and how these differ from the framework adopted in the present work. Notably, the thresholds delimiting the moderate‐risk and high‐risk categories in the current normative approach (15.32 and 17.24, respectively, on the age‐ and sex‐adjusted CHTQ Total score) are numerically close to the cut‐offs reported in the previous study (15 for females; 17 for males). This convergence, despite distinct methodological approaches, suggests that scores in the range of approximately 15−17 may represent a critical region along the CHT continuum associated with atypical trait expression. Importantly, however, the two sets of thresholds reflect different metrics and different decision targets. The prior cut‐offs were derived to predict the presence of psychopathology using sex‐stratified raw scores and represented an important first step toward operationalizing clinically meaningful thresholds. The present study extends this line of work by modeling age and sex as a continuous function, yielding a unified distribution of AS. Accordingly, these values should be viewed as partially convergent yet non‐equivalent indicators of higher levels of CHT.

To facilitate the practical and clinical application of the thresholds and cut‐offs proposed in the present study, a dedicated section within the Supporting Information (available in both Italian and English, Supporting Information S1: Appendix S1) provides the CHTQ instrument, detailed scoring instructions, age‐ and sex‐adjusted correction procedures, and percentile‐ and ES‐based interpretative grids. After computing raw scores and applying adjusted corrections, clinicians obtain the three adjusted indices (Total, IED, and MHS). These scores can be interpreted using ES categories, which function as a developmentally anchored risk‐stratification framework. Scores within the no/very low risk range (ES = 0–1) can be considered generally consistent with developmentally expected CHT levels. Low‐risk scores (ES = 2) may warrant contextualized monitoring, including consideration of developmental history, psychosocial stressors, and co‐occurring symptoms. Moderate to high‐risk classifications (ES = 3–4) indicate clinically meaningful deviation from normative expectations and support the need for structured assessment of mood symptoms, functional impairment, and longitudinal follow‐up, particularly when elevations are driven by the IED subscale. Importantly, ES categories are intended to complement, not replace, comprehensive clinical evaluation.

By contrast, ROC‐derived cut‐offs should be interpreted as criterion‐ and sample‐dependent discrimination thresholds anchored to bipolar‐spectrum status within the present study. Their most appropriate use lies within diagnostic pathways in which bipolarity already represents a clinically plausible hypothesis based on interview data and converging indicators (e.g., family history of bipolar disorder). In such contexts, ROC thresholds may serve as an additional quantitative reference to support diagnostic reasoning. However, their application as stand‐alone screening tools in unselected or normative populations should be approached with caution.

From a developmental and preventive perspective, the availability of age‐ and sex‐adjusted thresholds enhances the precision of risk identification by reducing both over‐ and under‐classification of normative affective variability. In clinical settings, the CHTQ may support the early recognition of adolescents presenting subthreshold or fluctuating affective features. In normative contexts, including school‐ or community‐based mental health initiatives, the instrument may help identify youths with emerging temperamental vulnerabilities and inform targeted, developmentally sensitive preventive strategies.

Despite the merits of this work, several limitations should also be acknowledged to gain an even clearer understanding of the results presented. First, the normative data in Study 1 were derived from a cross‐sectional, convenience‐based community sample. Although large, this sample may not fully represent the demographic and socio‐economic variability of the Italian adolescent population. For example, the sample does not include children aged 7–9 years or adolescent from 18 to 19 years. Additionally, raw‐to‐adjusted score transformations were modeled only on age and sex; other demographic or contextual variables (e.g., socio‐economic status, geographic area of residence) were not considered and may influence the expression of CHT. In this perspective, future studies will need to further verify the reference values identified using larger samples that are more representative of the Italian adolescent population. Regarding Study 2, clinical validation was restricted to adolescents with bipolar disorder, limiting the generalizability of findings to other clinical groups such as unipolar depression and ADHD. The modest AUC values also indicate that, although the CHTQ provides clinically relevant information, it should not be used as a standalone diagnostic tool and cannot replace structured clinical assessments. Moreover, the cross‐sectional nature of both studies prevents conclusions about the developmental stability of the identified risk thresholds or their prognostic utility. Future research should address these limitations by conducting longitudinal studies capable of evaluating the predictive validity of the age‐ and sex‐adjusted thresholds and their sensitivity to developmental change. Validation efforts should also be extended to broader and more diagnostically heterogeneous samples and replicated in cross‐cultural contexts to test the robustness of the normative values.

## CONCLUSIONS

In conclusion, the two complementary studies presented here provide the first set of normative benchmarks and clinical validation evidence for the Italian CHTQ in adolescence. By establishing age‐ and sex‐adjusted norms, delineating empirically informed risk thresholds, and examining their clinical relevance in a bipolar sample, this work advances the CHTQ from a purely descriptive instrument to a measure with clearer interpretative value in developmental and clinical contexts. The findings support the conceptualization of CHT as a transdiagnostic risk phenotype and underscore its potential contribution to early identification and preventive efforts in adolescent mental health. While the CHTQ should not be viewed as a diagnostic tool, its integration into clinical and community assessments may be particularly informative when interpreted alongside other converging risk indicators within a multifactorial model, helping to identify youths with emerging affective vulnerabilities who may benefit from closer monitoring or timely, developmentally tailored preventive interventions.

## AUTHOR CONTRIBUTIONS

**Anna Pezzella.** Conceptualization; data curation; formal analysis; writing—original draft. **Vincenzo Paolo Senese.** Conceptualization; writing—original draft; formal analysis; data curation. **Gabriele Masi.** Investigation; data curation. **Annarita Milone.** Data curation; investigation. **Giulia Mutti.** Investigation; data curation. **Pietro Buono.** Supervision; writing—original draft. **Gianluca Sesso.** Investigation; data curation. **Carmela Bravaccio.** Writing—review and editing; supervision. **Raffaella Iuliano.** Methodology; data curation. **Gennaro Catone.** Conceptualization; investigation; writing—review and editing. **Simone Pisano.** Conceptualization; writing—review and editing; methodology; supervision.

## CONFLICT OF INTEREST STATEMENT

The authors declare no conflicts of interest.

## ETHICAL CONSIDERATIONS

The present studies constitute secondary analyses of data previously collected and published. Ethical approval for the original studies was obtained from the respective institutional Ethics Committees, as also reported in the original publications, including the Ethics Committee of the University of Campania “Luigi Vanvitelli” (protocol no. 500, 29 April 2016) and the Institutional Review Board of Meyer Hospital (protocol no. 153/2017). An additional ethical approval was obtained from the Ethics Committee Campania 3 (protocol no. 125/2025, 09 July 2025). All procedures involving human participants were conducted in accordance with the Declaration of Helsinki, and written informed consent was obtained from the parents or legal guardians of all participants, along with assent from the adolescents.

## Supporting information

Supporting information S1

## Data Availability

The data that support the findings of this study are available from the corresponding author upon reasonable request.
