# Supplementary material for: Normative scores and clinical cut‐offs of the Cyclothymic–Hypersensitive Temperament Questionnaire in adolescence
Source: JCPP Adv. 2026 Apr 16:e70126. Online ahead of print. doi: 10.1002/jcv2.70126 (PMC13339036; doi:10.1002/jcv2.70126)
Supplement: Supplementary file 1 — Supporting information S1 [file JCV2-9999-e70126-s001.docx]

**Normative Scores and Clinical Cut-Offs of the Cyclothymic-Hypersensitive Temperament Questionnaire in Adolescence**

**Supporting Information**

**Appendix S1. CHTQ Instrument**

**Cyclothymic–Hypersensitive Temperament Questionnaire (CHTQ)**

**22-Item Self-Report English version**

Below you will find a series of statements describing personal characteristics, emotional reactions, and behaviours that may occur in everyday life. Please read each statement carefully and indicate whether it applies to you by selecting “**Yes**” or “**No**”.

|  | **Yes** | **No** |
| --- | --- | --- |
| 1. I often react intensely to minor upsets |  |  |
| 1. When I get upset, it’s very hard for me to calm down |  |  |
| 1. I have periods of irritation during which I can lose control |  |  |
| 1. I often experience intense emotions which I can feel all over my body (flushing, sweating, pounding heart) |  |  |
| 1. I experience all negative or positive emotions (both of sadness or joy) more intensely than others |  |  |
| 1. When watching a film, I often get overemotional (I can’t help crying, being scared, or laughing) |  |  |
| 1. My mood often changes without me knowing why |  |  |
| 1. Compared to my peers, when I get excited during the class recess, it is much harder for me to settle down upon return to the classroom |  |  |
| 1. I am very excited during a video game, and it’s very hard for me to calm down afterwards |  |  |
| 1. I experience rapid shifts in mood and energy |  |  |
| 1. At times I have a strong urge for risky or outrageous behaviour |  |  |
| 1. When I’m irritated, I can do stupid things I wouldn’t have done otherwise |  |  |
| 1. I can feel depressed for a few days and then be in a good mood again |  |  |
| 1. After a big stressful situation it can take several days for me to recover my composure |  |  |
| 1. I often need to attract the attention of others to me |  |  |
| 1. I am sometimes bubbling with energy, and at other times sluggish |  |  |
| 1. I know I have a tendency to get worked up, or to lose my temper too quickly when I’m frustrated |  |  |
| 1. I often experience severe, sudden emotions |  |  |
| 1. I can become strongly fond of a person I’ve just met |  |  |
| 1. Often I crave certain foods, cigarettes, alcohol or upper drugs |  |  |
| 1. I often daydream about things people consider unrealizable |  |  |
| 1. I alternate between feeling overly confident and feeling unsure and self-critical |  |  |

**Calculation of Raw Scores**

Responses are coded as follows: **“Yes” = 1; “No” = 0**

| **Total Score** | The total score is obtained by summing responses to all 22 items. |  |
| --- | --- | --- |
| **Subdimension IED**  *“Impulsiveness - Emotional Dysregulation”* | The score is obtained by summing responses to the following items:  1, 2, 3, 8, 9, 11, 12, 15, 17, 20 |  |
| **Subdimension MHS**  *“Moodiness - Hypersensitivity”* | The score is obtained by summing responses to the following items:  4, 5, 6, 7, 10, 13, 14, 16, 18, 19, 21, 22 |  |

**Calculation of Age- and Biological Sex–Adjusted Scores**

To obtain the adjusted scores for the total score and the two subdimensions, the clinician should identify, in the table below, the **correction factors** specific to each of the three dimensions according to the individual’s age and biological sex, and apply them directly to the corresponding raw scores by adding or subtracting the indicated value exactly as reported.

| **Total Score** | | | **Subdimension  IED** | | | **Subdimension  MHS** | | |
| --- | --- | --- | --- | --- | --- | --- | --- | --- |
| **Age** | **Sex** | **Correction factor** | **Age** | **Sex** | **Correction factor** | **Age** | **Sex** | **Correction factor** |
| 10 | F | -0.57 | 10 | F | -0.74 | 10 | F | +0.16 |
|  | M | +0.50 |  | M | +0.37 |  | M | +0.13 |
| 11 | F | -0.04 | 11 | F | -0.46 | 11 | F | +0.41 |
|  | M | +0.18 |  | M | +0.33 |  | M | -0.15 |
| 12 | F | +0.40 | 12 | F | -0.23 | 12 | F | +0.62 |
|  | M | -0.09 |  | M | +0.30 |  | M | -0.39 |
| 13 | F | +0.77 | 13 | F | -0.03 | 13 | F | +0.80 |
|  | M | -0.32 |  | M | +0.27 |  | M | -0.60 |
| 14 | F | +1.09 | 14 | F | +0.14 | 14 | F | +0.95 |
|  | M | -0.51 |  | M | +0.25 |  | M | -0.76 |
| 15 | F | +1.37 | 15 | F | +0.28 | 15 | F | +1.08 |
|  | M | -0.68 |  | M | +0.23 |  | M | -0.91 |
| 16 | F | +1.61 | 16 | F | +0.41 | 16 | F | +1.20 |
|  | M | -0.83 |  | M | +0.21 |  | M | -1.04 |
| 17 | F | +1.82 | 17 | F | +0.52 | 17 | F | +1.30 |
|  | M | -0.96 |  | M | +0.20 |  | M | -1.16 |
| **Adjusted Total Score:** | |  | **Adjusted IED Score:** | |  | **Adjusted MHS Score:** | |  |

**Interpretation of Scores**

**Percentiles**

Once the adjusted scores have been obtained, the individual can be positioned within the normative distribution using percentiles, which indicate the relative standing compared with age- and sex-matched peers.

| **Percentiles** | **Adjusted Total  Score** | **Adjusted IED  Score** | **Adjusted MHS Score** |
| --- | --- | --- | --- |
| 2.5^th^ | 1.2 | -0.3 | 0.8 |
| 5^th^ | 2.5 | -0.3 | 1.6 |
| 10^th^ | 3.8 | 0.5 | 2.8 |
| 20^th^ | 6 | 1 | 4 |
| 30^th^ | 7.5 | 1.7 | 5.2 |
| 40^th^ | 9 | 2.6 | 6 |
| 50^th^ | 10.09 | 3.03 | 6.76 |
| 60^th^ | 11.2 | 3.7 | 7.6 |
| 70^th^ | 12.5 | 4.7 | 8.2 |
| 80^th^ | 14.1 | 5.7 | 9 |
| 90^th^ | 15.6 | 6.7 | 10.2 |
| 95^th^ | 17 | 7.6 | 10.8 |
| 97.5^th^ | 17.8 | 7.7 | 11.2 |

**Clinical Risk Categories**

Adjusted scores can be classified into interpretative risk categories that allow a gradual and clinically meaningful reading of the Cyclothymic–Hypersensitive Temperament. These categories have no diagnostic value; rather, they represent progressive levels of temperamental vulnerability to be interpreted within a multifactorial model.

| **Risk Category** | **Adjusted Total  Score** | **Adjusted IED  Score** | **Adjusted MHS Score** |
| --- | --- | --- | --- |
| No risk | < 10.09 | < 3.03 | < 6.76 |
| Very low risk | 10.09 – 12.63 | 3.03 – 4.75 | 6.76 – 8.40 |
| Low risk | 12.64 – 15.31 | 4.76 – 6.66 | 8.41 – 9.79 |
| Moderate risk | 15.32 – 17.23 | 6.67 – 7.69 | 9.80 – 10.86 |
| High risk | ≥ 17.24 | ≥ 7.70 | ≥ 10.87 |

**Cyclothymic–Hypersensitive Temperament Questionnaire (CHTQ)**

**22-Item Self-Report Italian Version**

Di seguito troverai una serie di affermazioni che descrivono caratteristiche personali, reazioni emotive e comportamenti che possono presentarsi nella vita di tutti i giorni. Leggi con attenzione ciascuna frase e di indica se ti riconosci oppure no, selezionando **“Sì”** o **“No”**.

|  | **Sì** | **No** |
| --- | --- | --- |
| 1. Spesso reagisco intensamente a piccole controversie |  |  |
| 1. Quando mi arrabbio, è molto difficile per me calmarmi |  |  |
| 1. Ho periodi di irritabilità durante i quali posso perdere il controllo |  |  |
| 1. Spesso sperimento emozioni intense che si diffondono lungo tutto il corpo (arrossamento, sudorazione, cuore palpitante) |  |  |
| 1. Provo tutte le emozioni negative e positive (entrambe tristezza o gioia) più intensamente degli altri |  |  |
| 1. Quando guardo un film, spesso mi emoziono eccessivamente (non posso fare a meno di piangere, essere spaventato, o ridere) |  |  |
| 1. Il mio umore cambia spesso senza che io sappia il perché |  |  |
| 1. A confronto con i miei coetanei, quando mi eccito durante l'intervallo, è molto più difficile per me calmarmi una volta tornati in classe |  |  |
| 1. Mi faccio coinvolgere molto mentre gioco ai videogiochi, e è molto difficile per me calmarmi una volta finito |  |  |
| 1. Sperimento rapidi cambi di umore e energia |  |  |
| 1. Talvolta provo un forte impulso ad avere un comportamento rischioso o offensivo |  |  |
| 1. Quando mi irrito, posso fare cose stupide che altrimenti non avrei fatto |  |  |
| 1. Mi posso sentire depresso per alcuni giorni e poi essere nuovamente di buon umore |  |  |
| 1. Dopo una situazione altamente stressante, mi può servire qualche giorno per recuperare la mia tranquillità |  |  |
| 1. Spesso ho bisogno di attrarre l'attenzione degli altri su di me |  |  |
| 1. A volte sono pieno di energia, mentre altre volte mi sento spento |  |  |
| 1. So che a volte tendo ad arrabbiarmi, o a perdere il carattere troppo velocemente, quando sono frustrato |  |  |
| 1. Spesso sperimento intense e improvvise emozioni |  |  |
| 1. Posso legarmi fortemente a una persona appena conosciuta |  |  |
| 1. Spesso ho un desiderio incontrollato verso certi tipi di cibo, sigarette, alcool o droghe stimolanti |  |  |
| 1. Spesso sogno ad occhi aperti cose irrealizzabili o persone irraggiungibili |  |  |
| 1. Alterno sentimenti di eccessiva fiducia a sentimenti di insicurezza e di eccessiva autocritica |  |  |

**Calcolo dei punteggi grezzi**

Le risposte vengono codificate come segue: **“Sì” = 1**; **“No” = 0.**

| **Punteggio totale** | si ottiene sommando le risposte a tutti i 22 item |  |
| --- | --- | --- |
| **Sottodimensione IED**  *“Impulsività – Disregolazione Emotiva”* | si ottiene sommando le risposte agli item:  1, 2, 3, 8, 9, 11, 12, 15, 17, 20 |  |
| **Sottodimensione MHS**  *“Instabilità dell’umore – Ipersensibilità emotiva”* | si ottiene sommando le risposte agli item:  4, 5, 6, 7, 10, 13, 14, 16, 18, 19, 21, 22 |  |

**Calcolo dei punteggi corretti per età e sesso biologico**

Per ottenere i punteggi corretti del punteggio totale e delle due sottodimensioni, il clinico deve individuare nella tabella seguente i **fattori di correzione specifici** per ciascuna delle tre dimensioni in base all’età e al genere del soggetto e applicarli direttamente ai rispettivi punteggi grezzi, sommando o sottraendo il valore indicato così come riportato.

| **Punteggio totale** | | | **Sottodimensione  IED** | | | **Sottodimensione  MHS** | | |
| --- | --- | --- | --- | --- | --- | --- | --- | --- |
| **Età** | **Sesso** | **Fattore**  **di**  **correzione** | **Età** | **Sesso** | **Fattore**  **di**  **correzione** | **Età** | **Sesso** | **Fattore**  **di**  **correzione** |
| 10 | F | -0.57 | 10 | F | -0.74 | 10 | F | +0.16 |
|  | M | +0.50 |  | M | +0.37 |  | M | +0.13 |
| 11 | F | -0.04 | 11 | F | -0.46 | 11 | F | +0.41 |
|  | M | +0.18 |  | M | +0.33 |  | M | -0.15 |
| 12 | F | +0.40 | 12 | F | -0.23 | 12 | F | +0.62 |
|  | M | -0.09 |  | M | +0.30 |  | M | -0.39 |
| 13 | F | +0.77 | 13 | F | -0.03 | 13 | F | +0.80 |
|  | M | -0.32 |  | M | +0.27 |  | M | -0.60 |
| 14 | F | +1.09 | 14 | F | +0.14 | 14 | F | +0.95 |
|  | M | -0.51 |  | M | +0.25 |  | M | -0.76 |
| 15 | F | +1.37 | 15 | F | +0.28 | 15 | F | +1.08 |
|  | M | -0.68 |  | M | +0.23 |  | M | -0.91 |
| 16 | F | +1.61 | 16 | F | +0.41 | 16 | F | +1.20 |
|  | M | -0.83 |  | M | +0.21 |  | M | -1.04 |
| 17 | F | +1.82 | 17 | F | +0.52 | 17 | F | +1.30 |
|  | M | -0.96 |  | M | +0.20 |  | M | -1.16 |
| **Punteggio Corretto totale:** | |  | **Punteggio Corretto IED:** | |  | **Punteggio Corretto MHS:** | |  |

**Interpretazione dei punteggi**

**Percentili**

Una volta ottenuti i punteggi corretti, è possibile collocare l’individuo all’interno della distribuzione normativa mediante i percentili, che indicano la posizione relativa rispetto ai pari per età e genere.

| **Percentili** | **Punteggio corretto Totale** | **Punteggio corretto  IED** | **Punteggio corretto  MHS** |
| --- | --- | --- | --- |
| 2.5° | 1.2 | -0.3 | 0.8 |
| 5° | 2.5 | -0.3 | 1.6 |
| 10° | 3.8 | 0.5 | 2.8 |
| 20° | 6 | 1 | 4 |
| 30° | 7.5 | 1.7 | 5.2 |
| 40° | 9 | 2.6 | 6 |
| 50° | 10.09 | 3.03 | 6.76 |
| 60° | 11.2 | 3.7 | 7.6 |
| 70° | 12.5 | 4.7 | 8.2 |
| 80° | 14.1 | 5.7 | 9 |
| 90° | 15.6 | 6.7 | 10.2 |
| 95° | 17 | 7.6 | 10.8 |
| 97.5° | 17.8 | 7.7 | 11.2 |

**Categorie di rischio clinico**

I punteggi corretti possono essere classificati in categorie di rischio interpretative che permettono una lettura graduale e clinicamente significativa del Temperamento Ciclotimico–Ipersensibile. Tali categorie non hanno valore diagnostico, ma rappresentano livelli progressivi di vulnerabilità temperamentale, da interpretare all’interno di un modello multifattoriale.

| **Categorie di rischio** | **Punteggio corretto Totale** | **Punteggio corretto IED** | **Punteggio corretto MHS** |
| --- | --- | --- | --- |
| Assenza di rischio | < 10.09 | < 3.03 | < 6.76 |
| Rischio molto basso | 10.09 – 12.63 | 3.03 – 4.75 | 6.76 – 8.40 |
| Rischio basso | 12.64 – 15.31 | 4.76 – 6.66 | 8.41 – 9.79 |
| Rischio moderato | 15.32 – 17.23 | 6.67 – 7.69 | 9.80 – 10.86 |
| Rischio alto | ≥ 17.24 | ≥ 7.70 | ≥ 10.87 |

**Table S1.** *Descriptive statistics for adjusted scores of the CHTQ total score and its two subscales.*

| **Adjusted Scores** | **Mean (SD)** | **Median** | **Range** | **Skewness** | **Kurtosis** |
| --- | --- | --- | --- | --- | --- |
| CHTQ-Total | 9.95 (4.41) | 10.09 | [-0.61; 20.51] | -0.13 | -0.61 |
| CHTQ-IED | 3.39 (2.38) | 3.03 | [-0.52; 9.74] | 0.29 | -0.85 |
| CHTQ-MHS | 6.57 (2.76) | 6.76 | [-0.62; 12.39] | -0.32 | -0.54 |

**Table S2.** *Correction factors for computing adjusted scores of the CHTQ total score and its two subscales for each age and sex combination. The correction values have been pre-formatted with the appropriate sign; therefore, they should be subtracted from or added to the raw score exactly as reported.*

| **CHTQ Total** | | | **CHTQ IED** | | | **CHTQ MHS** | | |
| --- | --- | --- | --- | --- | --- | --- | --- | --- |
| **Age** | **Sex** | **Correction factor** | **Age** | **Sex** | **Correction factor** | **Age** | **Sex** | **Correction factor** |
| 10 | F | -0.57 | 10 | F | -0.74 | 10 | F | +0.16 |
|  | M | +0.50 |  | M | +0.37 |  | M | +0.13 |
| 11 | F | -0.04 | 11 | F | -0.46 | 11 | F | +0.41 |
|  | M | +0.18 |  | M | +0.33 |  | M | -0.15 |
| 12 | F | +0.40 | 12 | F | -0.23 | 12 | F | +0.62 |
|  | M | -0.09 |  | M | +0.30 |  | M | -0.39 |
| 13 | F | +0.77 | 13 | F | -0.03 | 13 | F | +0.80 |
|  | M | -0.32 |  | M | +0.27 |  | M | -0.60 |
| 14 | F | +1.09 | 14 | F | +0.14 | 14 | F | +0.95 |
|  | M | -0.51 |  | M | +0.25 |  | M | -0.76 |
| 15 | F | +1.37 | 15 | F | +0.28 | 15 | F | +1.08 |
|  | M | -0.68 |  | M | +0.23 |  | M | -0.91 |
| 16 | F | +1.61 | 16 | F | +0.41 | 16 | F | +1.20 |
|  | M | -0.83 |  | M | +0.21 |  | M | -1.04 |
| 17 | F | +1.82 | 17 | F | +0.53 | 17 | F | +1.30 |
|  | M | -0.96 |  | M | +0.20 |  | M | -1.16 |
